# RESOURCE UTILIZATION AMONG FAMILY CAREGIVERS: INDIVIDUAL AND NEIGHBORHOOD CHARACTERISTICS

**DOI:** 10.1093/geroni/igac059.1404

**Published:** 2022-12-20

**Authors:** Hyojin Choi, Kristin Litzelman, Autumn Harnish, Maija Reblin

**Affiliations:** University of Wisconsin-Madison, Madison, Wisconsin, United States; University of Wisconsin-Madison, Madison, Wisconsin, United States; University of Wisconsin-Madison, Madison, Wisconsin, United States; University of Vermont, Burlington, Vermont, United States

## Abstract

Resources in the community play a pivotal role in increasing well-being of both care recipients and caregivers and ensuring equitable access to needed resources is a crucial priority for policy and practice. Drawing on the Andersen Behavioral Model, this study explored the longitudinal relationship between individual and neighborhood factors and social resource utilization (i.e., paid caregivers, respite care, support group, training, financial help, or transportation) among family caregivers. Unpaid family caregivers for community-dwelling older adults were identified using data from the National Study of Caregiving and National Health and Aging Trends Study (Time 1: 2015, Time 2: 2017; n=616). Neighborhood characteristics were indicators of the Social Vulnerability Index and provider density at the census-tract level. Poisson regression was used to assess predictors of greater resource use (interpreted as incident rate ratios [IRR] with 95% confidence intervals [CI]). Nearly two-thirds of the sample reported using one or more services at each timepoint. Enabling factors were key predictors of resource use at Time 2, including income above 400% federal poverty level (IRR[CI]=1.53 [1.11,2.09]), better self-rated health (IRR[CI]=1.11 [1.01,1.22]), and resource use at Time 1 (IRR[CI]=1.64 [1.29,2.08]). Need-based predictors included greater frequency of personal care (IRR[CI]=1.13 [1.05,1.22] and longer care duration (IRR[CI]=0.99 [0.98,1.00]). Neighborhood factors were not associated with resource use in this analysis, nor were other need factors including caregiving burden. The findings highlight potential disparities in resource use by income, health status, or experience with systems navigation, with implications for policy and outreach.

